# Unilateral Renal Denervation for Refractory Hypertension in Aortic Dissection

**DOI:** 10.1016/j.jaccas.2026.107611

**Published:** 2026-03-25

**Authors:** Xian-Geng Hou, Jun-Shi Zhang, Guang-Mei Hu, Ying-Ying Zheng, Xiang Ma, Yi-Tong Ma, Xiang Xie

**Affiliations:** a Hypertension Department, Cardiovascular Center, The First Affiliated Hospital of Xinjiang Medical University, Urumqi, China; b Key Laboratory of Hypertension Research of Xinjiang Medical University, Urumqi, China; c Key Laboratory of High Incidence Disease Research in Xingjiang (Xinjiang Medical University), Ministry of Education, Urumqi, China; d Cardiac Rehabilitation Department, Cardiovascular Center, The First Affiliated Hospital of Xinjiang Medical University, Urumqi, China; e Cardiovascular Center, The First Affiliated Hospital of Xinjiang Medical University, Urumqi, China

**Keywords:** aortic dissection, refractory hypertension, renal denervation

## Abstract

**Backgroud:**

Refractory hypertension in patients with aortic dissection represents an extreme therapeutic challenge, as these patients are typically excluded from renal denervation (RDN) trials due to prohibitive procedural risks.

**Case Summary:**

A 40-year-old man with refractory hypertension complicated by a chronic DeBakey Type III (Stanford Type B) aortic dissection and abdominal aortic aneurysm presented with blood pressure of 171/98 mm Hg despite quintuple therapy. Unilateral right-sided RDN was performed using a multielectrode catheter, delivering 24 ablation lesions. Postprocedure, systolic blood pressure decreased by 30 mm Hg, enabling discontinuation of 2 antihypertensive agents.

**Discussion:**

This case demonstrates that unilateral RDN is safe and effective in patients with extreme vascular lesions where bilateral RDN is not suitable. The marked blood pressure response suggests heightened sympathetic drive in this population.

**Take-Home Message:**

RDN is also feasible for patients with aortic dissection, and even unilateral RDN can achieve a blood pressure–lowering effect. These findings are hypothesis-generating and require prospective validation.

## Visual Summary

**Figure:**
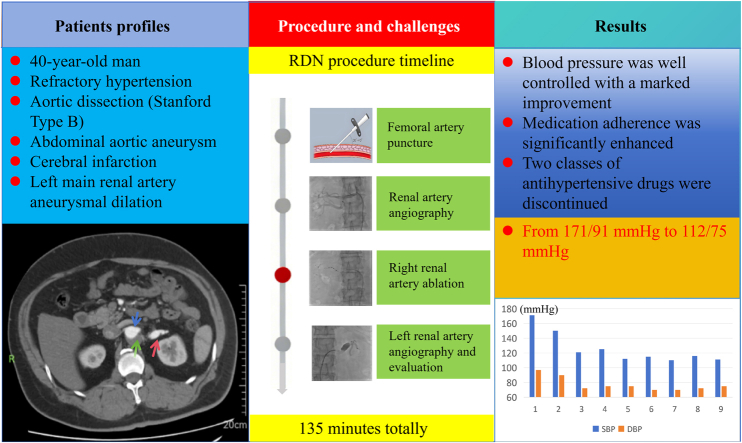

**Visual Summary:**
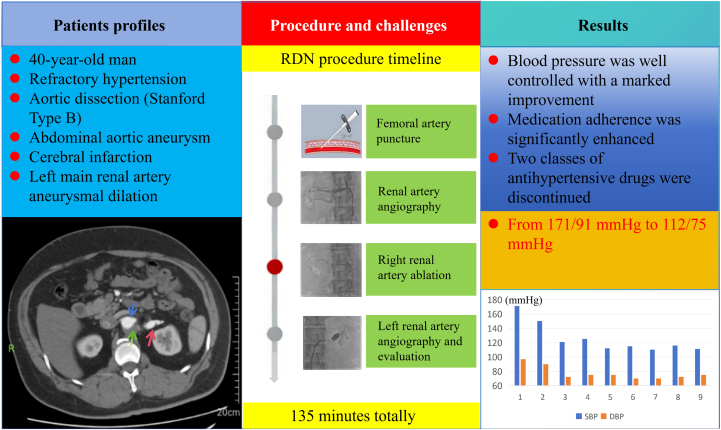
Unilateral Renal Denervation in Aortic Dissection Central illustration summarizing the case of a 40-year-old male with refractory hypertension and chronic DeBakey Type III (Stanford Type B) aortic dissection undergoing unilateral RDN. (Left) Patient profiles; (middle) procedure and challenges; (right) results and follow-up. DBP = diastolic blood pressure; RDN = renal denervation; SBP = systolic blood pressure.

## Background

Refractory hypertension (rHTN) affects 5% to 10% of hypertensive patients and confers a threefold increased risk of adverse cardiovascular events.^1^^,^^2^ Catheter-based renal denervation (RDN) has emerged as an important interventional option for patients whose blood pressure remains uncontrolled despite optimal medical therapy.^3^^,^^4^ However, major clinical trials have generally excluded patients with complex aortic disease—including aortic dissection and aneurysm—due to aberrant vascular anatomy and prohibitively high procedural risks.^5^^,^^6^ Consequently, there is limited evidence guiding RDN in this extreme-risk population.

We present a case of successful unilateral RDN in a patient with rHTN complicated by chronic aortic dissection and abdominal aortic aneurysm, highlighting the technical challenges and clinical decision-making process.

**Take-Home-Messages**

• Renal denervation is also feasible for patients with aortic dissection, and even unilateral renal denervation can achieve a blood pressure–lowering effect.
• Rigorous preprocedural computed tomography angiography assessment and multidisciplinary planning are essential for procedural safety.

## Case Presentation

A 40-year-old man was admitted with a 7-year history of hypertension. He initially presented in 2018 with sudden, severe back pain and was diagnosed with DeBakey Type III (Stanford Type B) aortic dissection and grade 3 hypertension, managed conservatively. His baseline antihypertensive regimen included sacubitril-valsartan 200 mg twice daily, nifedipine controlled-release 30 mg twice daily, metoprolol succinate extended-release 71.25 mg once daily, hydrochlorothiazide 12.5 mg once daily, and doxazosin 4 mg once daily. Three months prior to admission, he was hospitalized for acute cerebral infarction, with residual left-sided limb weakness. Additional medications included aspirin enteric-coated 100 mg daily, dl-3-n-butylphthalide 2 capsules 3 times daily, pitavastatin calcium 4 mg nightly, *Ginkgo biloba* extract 80 mg 3 times daily, and Naoxintong 3 capsules 3 times daily. His blood pressure had recently deteriorated, with office measurements reaching 171/98 mm Hg. He had a smoking history but denied other significant medical conditions.

**Figure 1:**
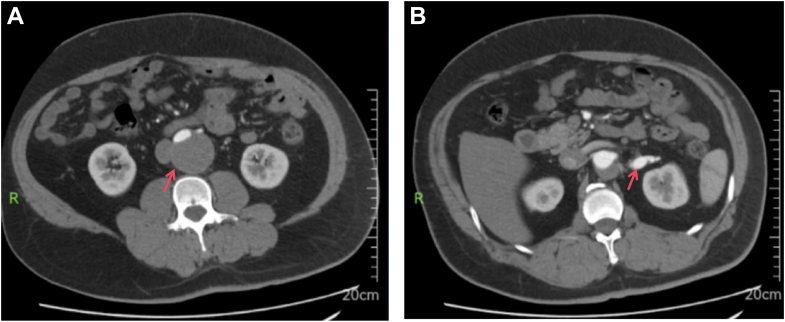
Preoperative Computed Tomography Angiography (A) Axial view showing chronic aortic dissection (Stanford Type B) extending to bilateral iliac arteries (arrow indicates compressed true lumen). (B) Axial view demonstrating aneurysmal dilation of left main renal artery (arrow).

Physical examination revealed blood pressure of 171/98 mm Hg and a pulse rate of 79 beats/min. Cardiac examination was unremarkable except for A2 >P2. No peripheral bruits, abdominal vascular bruits, or lower-extremity edema were present. Neurologic examination demonstrated left upper-extremity weakness (4/5). Aortic computed tomography angiography demonstrated chronic dissection extending from the L1 vertebral level to bilateral common iliac arteries, with organized thrombus in the false lumen (Figure 1A). The celiac trunk, superior mesenteric artery, and bilateral renal arteries arose from the true lumen, which was compressed but remained patent with adequate caliber to maintain organ perfusion. The abdominal aorta measured 5.0 cm at its widest point, indicating a concurrent abdominal aortic aneurysm. The left main renal artery exhibited fusiform aneurysmal dilation measuring 12 mm in diameter (Figure 1B). Right renal artery caliber was normal at 6 mm. Renal artery duplex ultrasound revealed reduced flow velocity in bilateral interlobar arteries and right renal hilar artery, consistent with chronic hypoperfusion. Echocardiography showed left atrial enlargement, left ventricular hypertrophy, and mild aortic regurgitation. Comprehensive workup excluded secondary causes of hypertension, including endocrine and renal parenchymal etiologies. Elevated liver enzymes, hypertriglyceridemia, and hyperuricemia were noted.

## Investigations Management

A multidisciplinary heart team concluded the patient had true rHTN. The persistent hypertension despite 5 agents warranted a device-based therapy. However, the dissected aorta introduced substantial risk requiring careful deliberation. After extensive discussion of benefits (blood pressure reduction, decreased cardiovascular events) and extreme procedural risks (dissection propagation, rupture, embolization), the patient strongly desired RDN and provided informed consent. Given the prohibitive risk of left renal artery ablation, unilateral right-sided RDN was planned.

**Figure 2:**
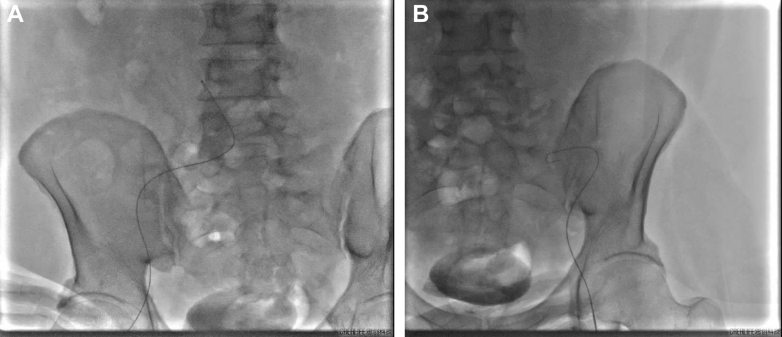
RDN Access Imaging Severe tortuosity of the bilateral femoral arteries resulted in difficulty in the access of puncture guidewires (A, right; B, left).

**Figure 3:**
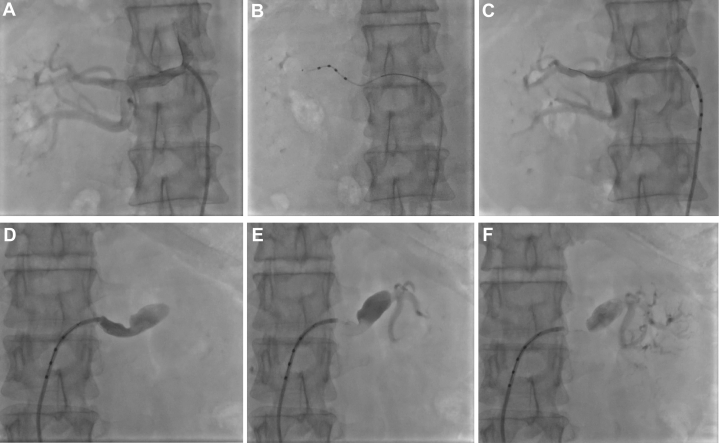
Intraprocedural Renal Angiography (A) Right renal artery preablation. (B) Spiral multielectrode catheter positioned in right renal artery branch. (C) Right renal artery postablation, showing no complications. (D to F) Left renal artery angiography showing aneurysmal dilation and slow flow.

The chronic dissection created a narrow, tortuous true lumen. Due to aortic dissection involving both iliac arteries, femoral artery puncture was challenging. Resistance was encountered when inserting a guidewire into the right femoral artery (Figure 2A), and the guidewire failed to pass through, prompting a switch to left femoral artery puncture. However, repeated attempts still proved unsuccessful, with the guidewire remaining impenetrable (Figure 2B). Ultimately, the guidewire was successfully inserted after changing the puncture site in the right femoral artery. To further confirm that the guidewire was in the true lumen, a 4-F vertebral angiographic catheter was gently advanced to the T12-L1 level, and angiography verified the catheter's position in the true lumen. Subsequently, after intravenous heparin 100 U/kg, a 6-F renal double-curve (RDC) guide catheter was positioned at the right renal artery ostium. Advancing the 6-F RDC guiding catheter required multiple careful manipulations under fluoroscopic guidance to avoid intimal flap injury. We used a “no-touch” technique, minimizing contact with the aortic wall. After RDC catheter positioning, connection to the pressure-monitoring system revealed that the systolic and diastolic blood pressures of the right renal artery were comparable to the peripheral blood pressure, indicating that luminal compression of the true lumen did not compromise blood supply to the right renal artery or its perfusion pressure. Under Thunder wire guidance, a multielectrode spiral radiofrequency ablation catheter was advanced to the distal secondary branches of the right renal artery. Using a distal-to-proximal, branch-to-trunk sequence, 24 radiofrequency ablations were performed. Postablation angiography demonstrated no renal artery spasm, dissection, contrast retention, or extravasation. After successful right-sided ablation, we assessed the left renal artery. Angiography confirmed the fusiform aneurysmal dilation with slow antegrade flow, consistent with computed tomography angiography findings (Figure 3). Given the risk of mural thrombus embolization and inadequate nerve ablation in dilated segments, we actively decided to abort left-sided treatment. Total procedural time was 135 minutes, and total contrast volume was 85 mL of iodixanol 320 mg/mL, with blood loss of 3 to 5 mL. No intraoperative complications occurred; no transfusions or implants were required.

## Outcome and Follow-Up

**Figure 4:**
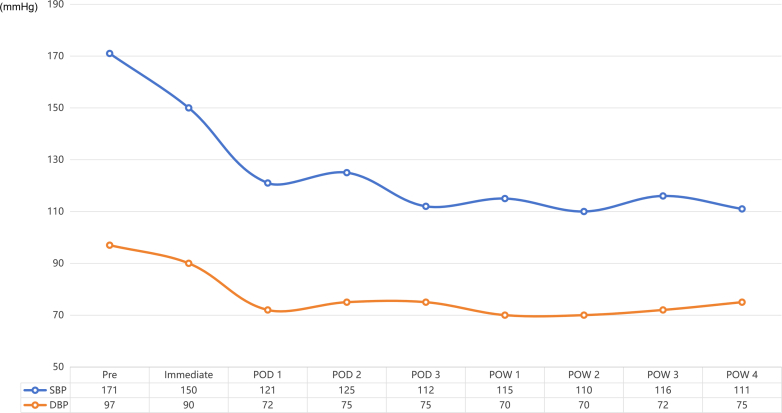
Blood Pressure Changes After Renal Denervation The blue line represents systolic blood pressure (SBP), and the orange line represents diastolic blood pressure (DBP). POD = postoperative day; POW = postoperative week.

As shown in Figure 4, immediate postoperative blood pressure fluctuated between 140 and 160/90 and 100 mm Hg. On postoperative day 1, blood pressure decreased to 121/72 mm Hg. On postoperative day 2, with stable blood pressure at 125/75 mm Hg, the regimen was adjusted to sacubitril-valsartan 200 mg twice daily, nifedipine controlled-release 30 mg twice daily, metoprolol succinate 71.25 mg once daily, aspirin 100 mg daily, and pitavastatin calcium 4 mg nightly. Hydrochlorothiazide and doxazosin were discontinued. On postoperative day 3, blood pressure was 112/72 mm Hg, and the patient was discharged. At 1-month follow-up, home blood pressure averaged 120/80 mm Hg during daytime and 110/70 mm Hg at night. Medication adherence improved, with no dizziness or hypotensive symptoms reported.

## Discussion

This case illustrates the therapeutic challenge when standard pharmacotherapy fails in the setting of life-threatening complications. The patient's chronic DeBakey Type III dissection with aneurysmal dilation mandated strict blood pressure control (<120/70 mm Hg) to limit false lumen expansion and prevent rupture, yet quintuple therapy failed to achieve target pressures. RDN emerged as a necessary therapeutic option in this high-risk patient. Unilateral (right-sided) RDN alone achieved dramatic, sustained blood pressure reduction from 171/98 mm Hg preoperatively to 110 to 120/70 to 80 mm Hg postoperatively, with successful medication simplification. This success provides valuable clinical insights for RDN practice in similar patients.

The most significant finding was the robust and sustained antihypertensive effect from unilateral RDN, contradicting the conventional belief that bilateral ablation is essential for efficacy. Previous sporadic reports have hinted at this potential, albeit in less complex contexts. Armaganijan et al^7^ described a patient who underwent only right-sided ablation due to intraoperative complications (iatrogenic contralateral renal artery dissection), achieving blood pressure improvement at 6 months (132/78 mm Hg vs 142/102 mm Hg baseline). Kostka-Jeziorny et al^8^ reported a patient who underwent only left-sided ablation due to intraoperative edema in the right renal artery wall causing 80% stenosis, demonstrating significant reductions in office, ambulatory, and central blood pressures at 12-month follow-up. The core challenges in these 2 prior cases were intraoperative complications, but the aortic system itself was intact. Our patient presented with an active DeBakey Type III aortic dissection and abdominal aortic aneurysm, representing an “unstable” vascular system. What remains unknown is the precise contribution of unilateral renal sympathetic innervation to the systemic sympathetic tone. Is extensive, multisegmental unilateral RDN sufficient to trigger significant systemic neurohumoral modulation? Or might unrecognized cross-renal neural regulatory pathways exist? These questions provide direction for future research and hope for patients in whom unilateral RDN is the only anatomically feasible option.

In addition, the aneurysmal dilatation of the left renal artery in this patient is most likely a chronic dissection-related medial degeneration secondary to aortic dissection, rather than fibromuscular dysplasia. We strongly recommend avoiding RDN at vascular segments with diffuse aneurysmal dilatation for the following reasons: 1) the depth of neural ablation makes it difficult to achieve the target due to arterial wall thinning; 2) there is a risk of mural thrombus formation and dislodgement; and 3) radiofrequency or ultrasound energy may induce aneurysm rupture. Alternative therapeutic strategies include strict blood pressure control and surveillance imaging follow-up; if clinically indicated, surgical revascularization or endovascular stent-graft implantation may also be adopted.

Limitations include a lack of ambulatory blood pressure–monitoring data, short follow-up duration, and concomitant medication changes that confound isolated RDN effect quantification. This single-case observation lacks ambulatory blood pressure–monitoring data and long-term efficacy assessment. Perioperative medication adjustments may confound quantification of RDN's independent effect. In addition, this represents a speculative, off-label application of RDN in a patient population excluded from major clinical trials. The findings are hypothesis-generating at best and require validation in dedicated prospective studies with robust safety endpoints before consideration for broader clinical practice.

## Conclusions

For patients with rHTN and complex aortic disease, RDN—including unilateral ablation—is a feasible, safe, and remarkably effective option after rigorous selection and multidisciplinary evaluation.

## Funding Support and Author Disclosures

This work was funded by Open Research Fund of Key Laboratory Co-constructed by Province and Ministry (SKL-HIDCA-2024-XXG3). The authors have reported that they have no relationships relevant to the contents of this paper to disclose.
